# Proceedings of Virginia State Dental Association

**Published:** 1875-04

**Authors:** 


### ARTICLE IV.

### *Virginia Dental Association.*—Continued.

THIRD DAY.—Thursday.

The subject of Mechanical Dentistry was resumed, and was discussed by Drs. Shanks, Wayt, Thompson, Sprinkle, Hunt and Smith.

Dr. Thompson thought it very important that dentists should have clean hands, and suggested the use of bicarbonate of soda and soap, when leaving the laboratory for the office.

Fifteen or twenty minutes were consumed in a conversational discussion of obtaining this desirable result.

Dr. Steel said this conversation was not only interesting and instructive, but was, though a small matter, quite an important one. Still we were trenching on the time which should be devoted to the discussion of the next subject in order—viz: That of Operative Dentistry. Before leaving that of Mechanical Dentistry, however, he wished to utter a word of warning, especially to the younger members of the profession. The subject of artificial denture had been so thoroughly ventilated, and reported in the papers so minutely, that attention would naturally be directed to it, and many inquiries made about and requisitions for the new base, and we were in danger of yielding too readily to the demand often made by our patients to extract slightly decayed natural teeth to be substituted for "prettier and more regular" artificial ones. The fight, therefore, for the preservation of the natural teeth would have to be renewed. He had said a thousand times he wished nothing cheaper than gold had ever been known, and could wish that artificial teeth were more expensive, that the inducement to sacrifice the natural teeth for artificial ones would be removed. Beautiful and useful as artificial teeth are, fulfilling as they do more of purposes for which they are designed than any other artificial organ, still he would not give one good, sound, natural tooth for a whole set of artificial ones. He related several cases in which he had by judicious filling saved the natural teeth after they had been condemned; had one case seventeen years standing, in which all but two or three of thirty odd fillings remained good to this day, he having but recently seen his patient, who when but a child, was told by another dentist her teeth could not be saved. His advice was therefore never to *recommend* the extraction of a tooth which could be saved, but if it was a hopeless case, then give your patient the benefit of the highest skill in supplying its loss.

On motion, the subject of Operative Dentistry was taken up and discussed.

Dr. Thackston stated that he would avail himself of the opportunity, while the subject of oxychloride fillings over the pulps was being discussed, to correct a misapprehension of his views as expressed at a former meeting of the Association. He did not on that occasion say or intend to convey the impression that the death and drying, or "*mummifying*" of dental pulps, sometimes observed after the application of "Guillois' Cement," was either a uniform or even usual result.

Dr. Mercer desired to ask his older brethren their views as to filling the natural teeth apart, and gave an account of what he thought was malpractice in his own mouth.

Dr. Henkel would not have the members think he favored plastic fillings as now in general use, because they were imperfect, and he never put them in with any assurance that they would last more than five days, though they often did last as many years.

Dr. Keesee gave an interesting account of some manipulations with oxychloride and Guillois' cement, and then filling with gold, but had been less successful with approximal cavities than with those on the grinding surface, and asked if the experience of others coincided with his. Dr. Burton had been more successful in these than in other cavities. Dr. Wayt's and Dr. Steel's experience was the same. Dr. Thackston's was that if the filling got wet it failed, if kept dry it would be successful. Dr. Burton used wax to keep the cavity dry. Dr. Thompson passed a heated burnisher over the filling. On motion the further discussion was closed.

The following gentlemen were elected officers for the ensuing year:

President, Dr. W. W. H. Thackston, of Farmville. Vice-Presidents—First, Dr. J. Hall Moore, Richmond; second, Dr. George B. Steel, Richmond; third, Dr. S. H. Henkel, Staunton. Treasurer, Dr. James F. Thompson, Fredericksburg. Secretary, Dr. George F. Keesee, Richmond.

Fourth Day.—Friday.

The Association met at the appointed hour, Dr. Thackston in the chair.

On motion, the discussion of "Operative Dentistry" was closed, and a report from the Committee on Dental Literature called for. Dr. Jeter, the chairman, had no written report, but called particular attention to the subject of children's teeth, the importance of which subject he discussed at some length, saying that great ignorance prevailed among the masses, and the people should be informed of their duty on this point.

Dr. Burton thought it so important a matter that he would like to give his views, which he was proceeding to do in an able and interesting manner when the President called attention to the fact that Dental Literature was under consideration, and that this was hardly to the point.

Dr. Burton then said he desired to call attention to Dr. White's treatise on "The Teeth," designed for patients; also to Dr. Garretson's Oral Surgery, and to another work by Dr. Meredith. He regretted that Dental Literature had not been of such a nature as to interest the people, hence they do not appreciate our services as they otherwise would. Many of the works were to voluminous. In giving information to our parents we should avoid as far as possible technicalities, using only such terms as are easily understood.

Dr. Moore added his testimony to the little books of Dr. White and Dr. Meredith as just what was wanted, especially as regards the information concerning the six year old molar. Even physicians often seemed ignorant in many instances about them, and frequently advised their extraction, to the detriment of the patient.

Dr. Thompson called attention to the fact that the publication of two dental journals had been commenced since the last meeting, making six periodicals now published. Also, a work on Dental Pathology by a German named Wedl.

Dr. Thackston endorsed what others had said, especially with reference to Dr. Garretson's work. He sincerely wished we had a periodical, which would be independent of any department. He exhorted the members to become writers and contributors to the journals. It would improve both writer and reader. We have the talent, and could make liberal contributions on the subjects of Operative Dentistry, Physiology, Pathology, Histology, Therapeutics and Microscopy.

Voluntary essays were next called for. At the request of the President, Dr. Steel read the paper received from Dr. Burkholder on "Atrophy of Teeth." It was heard with interest, and detailed a full and minute account of a most severe case of Atrophy, and was discussed by Drs. Moore, Burton, Wayt, Thackston and Keesee, and the thanks of the Association were tendered Dr. Burkholder.

A communication was read from Mr. Leroy Edwards, President of the Young Men's Christian Association, inviting the members to seats in the Christian Association Convention.

On motion of Dr. Steel, the by-laws were so amended as to make it the duty of the Executive Committee to provide for the entertainment of members attending the annual meetings.

On motion of Dr. Moore, complimentary resolutions and an appropriation of $50 were tendered Dr. Barnum for the donation to the profession of his rubber dam.

On motion, the following committee was appointed to revise the constitution and by-laws, and inquire into the expediency of having the same printed: Drs. Steel, Cowardin, and G. G. Wayt; to which was added the President and Secretary.

The thanks of the Convention and an appropriation of ten dollars were tendered the Young Men's Christian Association for use of hall; also, to Dr. Steel for valuable services rendered; also to the Petersburg, Chesapeake and Ohio, and Atlantic, Mississippi and Ohio Railroads, for reduced fares to delegates; also, to the officers of the Association for the faithful performance of their duties; also, to the newspapers for publishing proceedings.

Dr. George G. Wayt was elected a delegate to the Southern Dental Association, at Memphis; and Drs. Thackston, Moore, Henkel, Steel, Wood, Burton, and H. C. Jones, delegates to the American Dental Association, to meet at Niagara Falls.

The reception of a copy of Dr. Landon B. Edwards' *Virginia Medical Journal* was acknowledged.

Dr. J. S. Michard, was elected an active member of the Association.

Drs. L. M. Cowardin, John Mahony, and George G. Wayt were elected as the Executive Committee for the ensuing year.

On motion, the Association adjourned to meet in Richmond on the Tuesday after the second Monday in December, 1875.

The sessions of the Association were most harmonious, and a great deal of business was transacted, although our reports were somewhat curtailed.

---

